# *DAT1* Polymorphism Is Associated with Risk Taking in the Balloon Analogue Risk Task (BART)

**DOI:** 10.1371/journal.pone.0039135

**Published:** 2012-06-18

**Authors:** Rui Mata, Robin Hau, Andreas Papassotiropoulos, Ralph Hertwig

**Affiliations:** 1 Center for Cognitive and Decision Sciences, Department of Psychology, University of Basel, Basel, Switzerland; 2 Division of Molecular Neuroscience, Department of Psychology, University of Basel, Basel, Switzerland; 3 Division of Molecular Neuroscience, University Psychiatric Clinics, University of Basel, Basel, Switzerland; 4 Life Sciences Training Facility, Department Biozentrum, University of Basel, Basel, Switzerland; 5 Max Planck Institute for Human Development, Berlin, Germany; University of Pennsylvania School of Medicine, United States of America

## Abstract

Twin-studies suggest that a significant portion of individual differences in the propensity to take risks resides in people’s genetic make-up and there is evidence that variability in dopaminergic systems relates to individual differences in risky choice. We examined the link between risk taking in a risk taking task (the Balloon Analogue Risk Task, BART) and a variable number tandem repeat (VNTR) polymorphism in the 3′UTR of the dopamine transporter gene (*SLC6A3/DAT1*). Behavior in BART is known to be associated with activity in striatal reward-processing regions, and *DAT1* is assumed to modulate striatal dopamine levels. We find that carriers of *DAT1* alleles, which presumably result in lower striatal dopamine availability, showed more risk taking, relative to carriers of the alleles associated with higher striatal dopamine availability. Our analyses suggest that the mechanism underlying this association is diminished sensitivity to rewards among those who take more risks. Overall, our results support the notion that a behavioral genetic approach can be helpful in uncovering the basis of individual differences in risk taking.

## Introduction

A significant portion of individual differences in financial decision-making and risk-taking behavior can be attributed to genetic differences between individuals. This result is suggested by a number of twin-studies, with heritability estimates regarding behavioral measures of risk ranging from 20% to upwards of 60% [1]–[7]. Less is known, however, about the specific genes that underlie such behaviors. Our goal is to make further steps toward uncovering the potential genetic basis of risk taking by examining the role of a specific polymorphism of the dopamine transporter gene (*SLC6A3*; alternate symbol: *DAT1*). We examine its impact in a widely used and potentially clinically relevant behavioral measure of risk taking, the Balloon Analogue Risk Task (BART) [8].

Behavioral, neuroimaging, and genetic evidence converge in suggesting that genetic variability in dopaminergic systems is one potential source of individual differences in risky choice (see [9], for an overview). First, brain regions heavily innervated by dopaminergic pathways, such as the striatum and dorsolateral prefrontal cortex, have been shown to be implicated in decisions under risk [10]–[11]. Second, variation in dopaminergic function has been related to behavioral measures of risk taking [6], [12] and to personality traits that appear to accompany risk taking, such as extraversion [13], impulsivity [14], and novelty seeking [15]. Finally, there are links between genetic variation in dopaminergic genes and real-world risk-taking and addiction [16]–[17].

One candidate gene that may account for a portion of the variation in risk-taking behavior is the dopamine transporter gene (*DAT1*). It codes for a dopamine transporter protein that is expressed abundantly in the striatum and is responsible for regulating the dopaminergic release into the extracellular space by recapturing dopamine into presynaptic terminals [18]. The gene, which includes 15 exons, harbors a variable number of tandem repeats (VNTR) polymorphism in its 3′UTR. The 40-bp VNTR element is repeated between 3 and 13 times but occurs with greatest frequency in the 9- and 10-repeat forms [19]–[21].

With regard to the functional effects of *DAT1* on dopaminergic function, a number of studies suggest that the *DAT1* 9-repeat allele, relative to the 10-repeat allele, is associated with reduced expression of dopamine transporter protein, resulting in relatively increased extrasynaptic striatal dopamine levels in the former [22]–[25]. Some studies, however, have reported the opposite direction of effect [26]–[28], whereas others could not find any functional effects of *DAT1* [29]–[30]. Despite this mixed picture, on the whole there is evidence for a role of *DAT1* on reward processing that is consistent with the thesis that the 9-repeat allele is associated with increased striatal dopamine levels relative to the 10-repeat allele [31]–[35]. Some results from other domains–for instance, investigations of implicit learning [36] and cognitive control [37]–are also in line with this effect.

How can people’s propensity to take risks be gauged in the laboratory? Although numerous tasks have been designed to determine people’s risk-taking propensity, many have been criticized for being too artificial and lacking external validity [38]. One exception is the Balloon Analogue Risk Task (BART). It embodies a naturalistic setting that has been shown to be predictive of real-world risk-taking [8]. During the BART, a balloon appears on a computer screen and participants are asked to inflate it by pressing a button on the screen. Each successful pump, simulated on the screen, results in a fixed amount of money for this round. Participants are told that the balloon will explode somewhere between the first pump and the balloon filling up the screen. Should they pump until the balloon bursts, they lose all rewards collected in this trial. Thus, on each trial, participants have to decide when to stop pumping and collect their rewards.

Risk taking as observed in the BART is correlated with (a) self-reported measures of impulsivity and sensation seeking [8], [39]–[40], (b) self-reported real-world risk behaviors such as smoking [41]–[42], unsafe sex [43], and risky drug use [8], [44], and (c) another behavioral measure of risk, the Iowa Gambling Task (once behavior is decomposed through cognitive modeling) [45]. A number of studies have demonstrated a significant contribution of both frontal and striatal brain regions to risk behavior in the BART. Concerning frontal contributions, functional imaging studies have shown involvement of dorsal lateral prefrontal cortex in the BART [46]–[48]. Also, an EEG study found that frontal cortex activity was related to balloon bursts and that smaller amplitudes in response to balloon bursts were associated with a family history of alcohol problems [49], suggesting that heritable factors related to sensitivity to losses may underlie both risk taking in the BART and alcoholism. Another study showed that transcranial direct current stimulation (tDCS) of the dorsolateral prefrontal cortex leads to reduced pumping in the BART, thus suggesting a causal role for frontal structures in regulating risk taking [50]. Administering a version of the BART to rodents, researchers found that temporary inactivation of a region homologous to the human dorsolateral prefrontal cortex resulted in increased variability in behavior and sub-optimal performance in the BART but suggested that these frontal regions may exert their influence “through direct regulation of specific striatal zones” [51]. In line with this conjecture, other findings suggest an involvement of mesolimbic areas in risk taking in the BART. A functional magnetic resonance imaging (fMRI) showed that risk taking in the BART is associated with robust activation in the ventral and dorsal striatum and anterior insula [46]. Other studies found similar results with increased dorsal striatum activation being linked to fewer pumps on the BART [47]. Also, a comparison of Parkinson’s disease patients with and without impulse control symptoms has showed that patients with impulse control deficits showed significantly diminished BOLD activity in the ventral striatum during engagement with the BART [48], thus suggesting that striatal function is linked to risk taking in the BART and impulsivity more generally.

Based on these results, in particular those suggesting an important role of mesolimbic structures in the BART, we derived two hypotheses concerning the role of dopaminergic function on individual differences in risk taking in the BART. According to the *reduced-reward-sensitivity* hypothesis, individual differences in risk taking are caused by differences in sensitivity to rewards. Specifically, it has been suggested that diminished striatal dopamine levels, lowering reward sensitivity, can lead individuals to seek more monetary or food rewards than people with higher levels [52]–[53]. Consequently, in the BART, low reward sensitivity could lead to increased pumping and thus greater risk taking–a link that is consistent with decreased striatal activation being associated with more pumping in the BART [47]–[48].

Alternatively, however, more risk taking in the BART may not be due to reduced reward sensitivity but to more indifference toward losses. Specifically, according to the *reduced-loss-sensitivity hypothesis*, interindividual differences in risk taking may be driven by differential sensitivity to those losses that materialize once a balloon bursts [49]. Some researchers have speculated that individual differences in loss aversion are related to naturally occurring differences in dopaminergic function [11]. To the extent that reduced levels of striatal dopamine bring about reduced sensitivity to losses, individuals with lower levels may react less strongly to balloon bursts (losses) and, consequently, are able to tolerate more risk (i.e., more pumps). In sum, both hypotheses suggest that reduced striatal dopamine availability plays a role in risk taking in the BART. The diminished-reward sensitivity account attributes more risk taking in the BART to reduced sensitivity to rewards. The diminished-loss sensitivity account, in contrast, suggests a direct link between more risk taking and reduced reactivity to losses. Put simply, the former account portrays the risk takers as those who gain less utility (satisfaction) from a unit of reward than more risk-averse people; the latter account envisages the risk takers as suffering from less disutility from a given loss than more risk-averse people.

Our study examines the extent to which dopaminergic function can modulate risk taking by linking a genetic polymorphism (*DAT1* VNTR) thought to modulate striatal dopamine function to behavior in the BART. In the process, we will aim to determine whether diminished reward or loss sensitivity accounts for a higher propensity to take risks.

## Methods

### Participants

The study was approved by the ethics board of the canton Basel (Ethikkomission beider Basel; www.ekkb.ch) and all participants provided written informed consent before participating in the study. Three hundred and sixty-nine adults participated in the study. A power analysis was performed that suggested that upwards of 300 participants would be needed to achieve acceptable power (>.80; e.g. t-test between roughly equal-sized groups, α = .05, Cohen’s *d* = .25). Based on this analysis we aimed to sample at least 300 participants and used the end of the academic year as a stopping rule. As a consequence, data collection spanned two consecutive academic years (2008/2009 and 2009–2010) and was stopped at the end of the second academic year. Data were collected in Basel, Switzerland. The majority of participants were students at the University of Basel, a culturally diverse but predominantly Caucasian population. Students were recruited through paper postings and the psychology department’s online recruitment system. From the available saliva samples, we were able to genotype a subset of individuals (*N* = 331). In addition, visual inspection of the data for individual participants suggested that a few were not engaged in the task as evidenced by pumping only once on every trial (the minimum allowed), pumping only once after a few trials, or always pumping until the balloon exploded on every trial. We excluded participants in a principled manner by excluding 9 outliers whose average number of pumps/adjusted pumps in the BART exceeded ±3 SD of the group means. The overall pattern of results does not change, however, when these individuals are included in the analyses reported below. The final sample was composed of 322 participants (88 men; age range = 18–55, *M* = 23.8 years, *SD* = 6.2). No participants reported neurological or psychiatric problems or taking medication targeted at the dopaminergic system.

### Genetic Analysis

DNA was extracted from Oragene® DNA sample collection kits (DNA Genotek Inc., Ontario, Canada). The 40-basepair VNTR polymorphism in the 3′ UTR of *DAT1* was genotyped by PCR using the following primers; forward: 5′ -TGTGGTGTAGGGAACGGCCTGAG-3′ reverse: 5′ -CTTCCTGGAGGTCACGGCTCAAGG-3′ using standard PCR settings. The PCR products were visualized on a 2% agarose gel. The sizes of the various repeat alleles were: 7-repeat (360 bp), 8-repeat (400 bp), 9-repeat (440 bp), 10-repeat (480 bp), and 11-repeat (520 bp).

A minority of participants in our sample showed a 9-repeat/9-repeat genotype (9/9; *n* = 20). Most showed 9-repeat/10-repeat (9/10; *n* = 139) or 10-repeat/10-repeat (l0/10; *n* = 167) genotypes, and still another small group showed a 10-repeat/11-repeat genotype (10/11; *n* = 4). Genotypes were in Hardy-Weinberg equilibrium (*P* = 0.37). In our analysis, the following genotype groups were compared: one group consisting of 9-repeat carriers (9 carriers; *n* = 159) and another group consisting of non-carriers of the 9-repeat allele (i.e., 10-repeat homozygotes and 11-repeat carriers [10/10 and 11 carriers]; *n* = 171).

### Balloon Analogue Risk Task

Participants were faced with a series of 30 balloons (i.e., trials) on the computer screen. In each trial, participants sequentially pressed a button on the screen to inflate the balloon. The sequence ended when a person chose to stop or the balloon exploded. Participants did not complete practice trials or were informed about the probability structure governing the balloons’ exploding. For example, participants would have to learn from experience that the probability of the balloon exploding increased with each pump. However, participants were told that at some point each balloon would burst and that this explosion could occur at any point from the first pump to when the balloon had expanded to fill the entire screen.

Each successful click increased the participants’ temporary payoff but involved the risk that the balloon would explode. Participants could decide when to stop pumping the balloon and collect the payoff from their temporary account. If the balloon exploded following a pump, the participant earned nothing for that trial. If a person stopped inflating the balloon before it exploded, the money earned was transferred to a permanent bank depicted on the screen. Then a new balloon appeared and the next trial began.

The point at which a balloon would burst was determined randomly for each trial by drawing a number between 1 and 128 from a uniform distribution to represent the threshold number of pumps at which the balloon would burst on that trial. The resulting probability that a balloon will burst given a number of pumps (*pumps*) can be described as follows [8]:
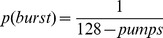
(1)Equation 1 implies that the probability of a balloon bursting is a function of the number of pumps. For example, the probability of the balloon bursting is very small after pumping once, *p*(burst) = 1/(128–1) = 1/127≈.008. After 127 successful pumps, however, it is certain that one more pump will cause the balloon to burst, *p*(burst) = 1/(128–127) = 1/1 = 1. Participants received .01 Euro for each successful pump (i.e., not causing an explosion). The strategy promising the highest expected gain in the task is to pump 64 times in each of the 30 trials: this number represents the average burst point and would result in an explosion on 15 of 30 trials on average.

### Procedure

Participants first read and signed the informed consent form. Then, participants completed a paper questionnaire concerning their age, sex, and medical status, followed by computerized testing which included the 30 BART trials, and finally provided a saliva sample for genotyping.

## Results

**Figure 1:**
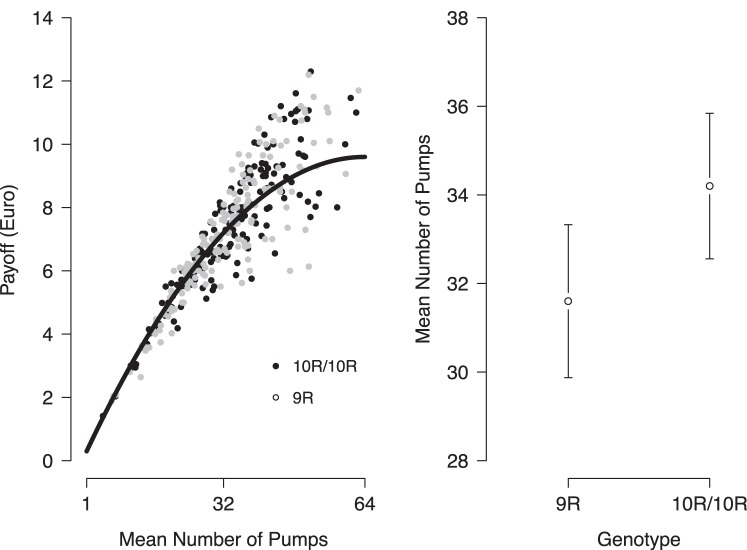
Left: Payoff in the Balloon Analogue Risk Task (in Euro) by mean number of pumps for 9-repeat carriers (9R) and 10-repeat homozygotes and 11-repeat carriers (10R/10R). The black line represents the expected value of the respective mean number of pumps.Right: Mean number of pumps and respective 95%CIs for 9-repeat carriers (9R) and 10-repeat homozygotes and 11-repeat carriers (10R/10R).

Table 1 presents summary measures of participants’ performance in the BART, separately for the 9-repeat carriers and the 10/10 and 11-repeat carriers. Across trials, the 9-repeat carriers pumped significantly (*p* = 0.031) fewer pumps and thus earned less money than the 10/10 and 11-repeat carriers. The effect size was small (Cohen’s *d* = 0.24). Figure 1 depicts participants’ payoff as a function of average number of pumps as well as the mean number of pumps as a function of genotype. It shows, as expected, that payoff increases non-linearly as the average number of pumps approaches the optimal value in the task (64 pumps; see task description above). There is, of course, considerable variability in payoff for a given number of pumps because of the probabilistic nature of bursts in the BART. In addition, it shows that 10R/10R and 11R tended to pump more than the 9R counterparts. Overall, these results provide the first demonstration that there is a link, albeit small in magnitude, between the *DAT1* polymorphism and risk taking in the BART, with 9-repeat carriers showing less risk taking relative to 10/10 and 11-repeat carriers.

**Table 1:** Means (SD) for demographic and Balloon Analogue Risk Task (BART) variables as a function of *DAT1* Groups.

Variables	9-repeat carriers	10/10 and11-repeat carriers	κ^2^ or t statistic	p-value	Cohen’s d
*n* (males)	156 (44 ♂)	166 (44 ♂)	–	–	–
Right Handedness	142 (91%)	149 (90%)	0.06	.81	–
Age	24.1 (6.9)	23.4 (5.2)	1.04	.30	0.12
Pumps	31.6 (11.0)	34.2 (10.8)	2.17	.031	0.24
Adjusted Pumps	35.7 (14.1)	38.9 (13.9)	2.01	.045	0.23
Bursts	9.3 (3.5)	9.9 (3.5)	1.56	.120	0.17
Payoff	7.0 (2.1)	7.5 (2.1)	1.96	.050	0.24

Note. Adjusted Pumps = Pumps in unexploded balloons.

Having established this link, we can now ask whether increased risk-taking (i.e., pumping) in the 10/10 and 11-repeat carriers is brought about by diminished sensitivity to losses or by diminished reward sensitivity. To discern between these two possibilities we tested for effects of *DAT1* genotype on risk adjustment after losses (i.e., balloon bursts). The analysis’ rationale is that if increased risk taking were related to insensitivity to losses, differential risk adjustment following negative outcomes would emerge as a function of genotype.

We employed a multilevel, mixed-effects modeling approach to simultaneously model individual and group differences in pumping frequency and sensitivity to losses (i.e., balloon explosions). Mixed-effects models allow researchers to simultaneously consider standard fixed-effects, but also covariates bound to the items (e.g., trial type: burst vs. non-burst) or participants (e.g., genotype grouping, age, sex). Crucially, these techniques allowed us to model learning (trial) effects as well as local dependencies between successive trials without requiring prior averaging across participants or items that eliminates potentially informative variation in the data and reduces statistical power [54].

We first conducted an initial screening of the amount of within-person variability in the pumping data using the intraclass correlation obtained from an unconditional means model in which the residual variance was significant. The analysis indicated that around one third of the total variance in pumping behavior was located within persons (intraclass correlation = .28). Moreover, the mean reliability was good (.92), suggesting it warranted conducting a multilevel analysis. We then used the following set of equations to model pumping behavior in the BART:
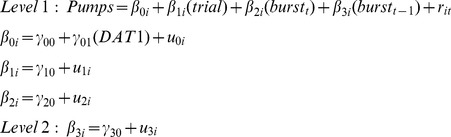



In Level 1, pumping of participant *i* on trial *t* is a function of (a) the intercept (*β*_0*i*_), (b) trial number (*β*_1*i*_; 1 to 30), (c) whether the trial is a burst trial or not (*β*_2*i*_; 0 = no burst, 1 = burst), (d) whether the previous trial was a burst trial or not (*β*_3*i*_; 0 = no burst, 1 = burst), (v) and the residual (*r_it_*). The equation thus captures for each single individual a number of potential effects of interest. First and foremost, the effect of *learning*: Participants typically pump less than the optimal amount and increase their pumping with experience (i.e., across trials) [8]. Second, the equation captures differences between trials in which the balloon explodes and those in which it does not as is commonly done by distinguishing between average number of pumps and average number of pumps in unexploded balloons (*burst_t_*) [8]. Finally, the equation accommodates the possibility that participants adjust their risk taking as a function of the previous trial (*burst_t−1_*). To our knowledge, reactivity to burst trials has not been investigated in the past, so our analysis represents an important pioneering contribution to understanding trial-by-trial performance in the BART.

In the Level 2 equations, γ_00_, γ_10_, γ_20_, and γ_30_, represent mean weights given to each factor represented in Level 1 and the respective residuals, u_0*i*_, u_1*i*_, u_2*i*_, u_3*i*_. In addition, we added *DAT1* (9-repeat carriers = 0; 10/10 and 11-repeat carriers = 1) as a predictor at the level of the intercept to capture mean level differences in pumping as a function of *DAT1* genotype (Model 1).

Crucially, we also tested a number of additional models that considered additional effects of *DAT1*, including an interaction between *DAT1* and the adjustment of risk as a function of the previous trial (Model 2: *β*_3*i*_ = γ_30_+γ_31_ (*DAT1*)+u_3*i*_). The rationale for including an effect of *DAT1* at this level is that observing an effect of genotype would suggest that *DAT1* moderates risk taking through differences in reactivity to losses, as suggested by the diminished-loss-sensitivity hypothesis. We also considered further interactions between *DAT1* and trial to account for possible differences in learning rates as a function of genotype (Model 3: *β*_1*i*_ = γ_10_+γ_11_ (*DAT1*)+u_1*i*_), and bursts (Model 4: *β*_2*i*_ = γ_20_+γ_21_ (*DAT1*)+u_2*i*_). Finally, we considered a model that included the available control variables (age, sex, handedness; Model 5). We estimated parameters for all models using the MCMCglmm package for R [54], and used the chi-squared distributed Deviance Information Criterion (DIC) as a measure of fit [55], [56].

As expected, Model 1 (DIC = 81718), provided a better fit to participants’ data relative to a baseline model (unconditional means model; DIC = 82202), ΔDIC = 81718–82202 = −484, *df* = 13, *p*<.001. In turn, Model 2, which included the possibility that *DAT1* polymorphism moderates the impact of a loss in the previous trial, did not prove considerably better than Model 1, ΔDIC = −1.4, *df* = 1, *p* = .24. This finding suggests that *DAT1* does not moderate reactivity to losses. The other models also did not prove better than Model 1, Model 3, ΔDIC = −0.4, *df* = 1, *p* = .53, Model 4, ΔDIC = −1.1, *df* = 1, *p* = .29. Finally, controlling for age, sex, and handedness, Model 5, ΔDIC = 5.8, *df* = 3, *p* = .12, did not change the pattern of results already identified in Model 1.

**Figure 2:**
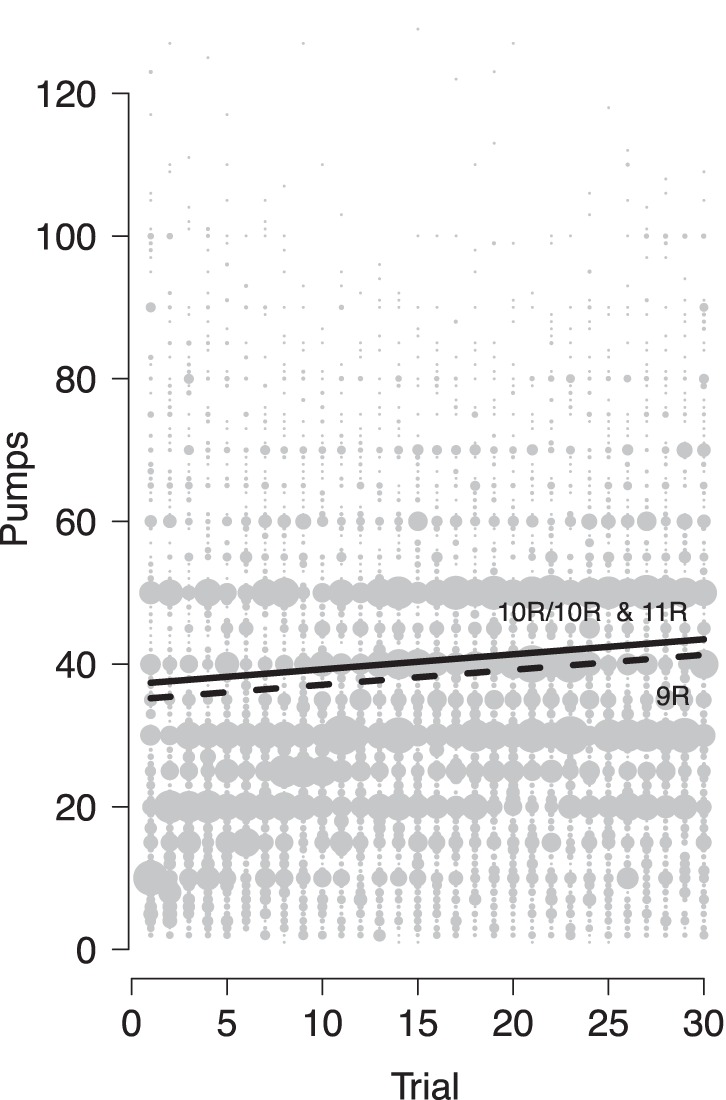
Average pumps on self-terminated trials as a function of trial number for 9-repeat carriers (9R; dashed line) and 10-repeat homozygotes and 11-repeat carriers (10R/10R & 11R; solid line), as estimated from multilevel modeling (see text). The diameter of the circles is proportional to the number of participants that gave the respective number of pumps on that trial.

The parameter estimates, the respective confidence intervals, and *p* values for the fixed effects for Model 1 parameters are given in Table 2 and the main effect of genotype is depicted in Figure 2. The results indicate that our participants showed the typical behavioral pattern found in previous studies: with, on average, 33 pumps given initially they remained far below the optimal 64 pumps [8]. Nevertheless, there was evidence of learning with participants increasing their pumping by, on average, one pump every 5 trials (leading to an average of 39 pumps in the final of 30 rounds). In addition, there was an effect of *burst*, with about 11 fewer pumps being made in those trials in which balloons exploded relative to those in which it did not. Furthermore, a burst in the previous trial (*burst_t−1_*) affected the subsequent trial, with, on average, 4 fewer pumps in the trial following a burst trial. This result is the first demonstration that participants adjust their pumping behavior on the basis of bursts on a trial-by-trial basis. Finally, there was an effect of *DAT1*, with 10/10 and 11-repeat carriers engaging in between 2 to 4 pumps more per balloon relative to 9-repeat carriers.

## Discussion

Our findings suggest that *DAT1* polymorphism is associated with risk taking in a widely used and potentially clinically relevant task, the Balloon Analogue Risk Task [8]. Specifically, carriers of *DAT1* alleles, which presumably result in lower striatal dopamine availability (10-repeat homozygotes and 11-repeat carriers), engaged in more risk taking relative to those with higher striatal dopamine availability (9-repeat allele carriers). Based on our analysis of the carriers of different alleles to losses, we conclude that more risk taking in the BART appears to be associated with diminished reward sensitivity but not sensitivity to losses. Specifically, individuals with lower striatal dopamine availability tend to seek higher rewards (and therefore tolerate more risk). This notion has also been proposed to explain individual differences in gambling [52] and weight-gain [53]. For example, genetic risk for reduced signaling of dopamine-based reward circuitry and consequently low striatal response to food intake (reward) predicts future weight gain [57].

An alternative interpretation of our findings is that lower striatal dopaminergic availability negatively influence cognitive control processes, such as the ability to inhibit pumping in the BART. Our data cannot exclude this alternative explanation but the pattern of results regarding the link between DAT1 and cognitive control is mixed [37], [58], thus leading us to favor the diminished-reward sensitivity hypothesis. Future work that links DAT1 status to both BART and cognitive control measures such a Go/NoGo performance could test between these two alternative explanations.

**Table 2:** Parameter Estimates from the Multilevel Regression Modeling for Model 1 (see text).

Fixed Effects	Coefficient	−95% CI	+95% CI	*p*
Intercept (γ_00_)	33.39	32.51	34.35	<.001
Trial (γ_10_)	0.19	0.11	0.26	<.001
Burst*_t_* (γ_20_)	−11.73	−12.43	−11.02	<.001
Burst*_t–1_* (γ_30_)	−4.10	−5.10	−3.16	<.001
*DAT1* (γ_01_)	2.82	1.63	3.97	<.001

Our investigation has a number of limitations that deserve to be addressed in the future. First, the genetic association reported herein has to be replicated in independent studies and is therefore considered to be preliminary. Second, we focused on a single genetic polymorphism, *DAT1* VNTR, which is only one of several polymorphisms known to regulate striatal dopamine levels [9]. Moreover, there are important frontal contributions to behavior in the BART [46]–[50], such as those from the dorso-lateral prefrontal cortex where the dopamine transporter is not significantly expressed. Consequently, future investigations should try to assess a more comprehensive number of polymorphisms related to frontostriatal function, including COMT, DARPP-32, DAT1, DRD2, and DRD4, among others [9]. Assessing multiple polymorphisms will require making assumptions about possible interactions between genes [34]. Nevertheless, establishing such profiles could prove fruitful in combination with imaging methods to observe to what extent brain activation varies as a function of genetic makeup [33]. Alternatively, future research could employ a genome-wide association study-design to identify possible new molecular pathways associated with risk taking. However, such studies must include larger samples (*n*>1000). One important advantage of conducting such large-scale studies is the possibility of considering theoretically meaningful assessments of less frequent genotype groups, such as the infrequent 9-repeat-homozygous group for DAT1 that represented a minority in our sample, and were thus collapsed with the 9-repeat-carriers to ensure acceptable statistical power in our analyses.

Finally, one may also consider a change to our probabilistic implementation of the BART. Because of its random probability structure, the BART will produce considerable differences in payoff notwithstanding similar pumping behavior. This structural feature of the task contributes to its realism but it also increases random variability in individuals’ payoff. Consequently, one may want to further study the impact of dopaminergic function on risk taking by providing all individuals with the same set of bursting points across trials.

In conclusion, our findings suggest that a genetic polymorphism of the dopamine transporter gene, *DAT1*, is linked to risk taking in a task that has been shown to be associated with self-reported clinical risk taking. Moreover, our results suggest that the mechanism underlying this association is diminished sensitivity to reward (and not diminished sensitivity to losses). Finally, our results support the notion that the behavioral genetic approach can be helpful in uncovering the basis of individual differences in risk taking.
